# Fortification of table olive packing with the potential probiotic bacteria *Lactobacillus pentosus* TOMC-LAB2

**DOI:** 10.3389/fmicb.2014.00467

**Published:** 2014-09-03

**Authors:** F. Rodríguez-Gómez, V. Romero-Gil, P. García-García, A. Garrido-Fernández, Francisco N. Arroyo-López

**Affiliations:** Department of Food Biotechnology, Instituto de la Grasa, Consejo Superior de Investigaciones CientíficasSeville, Spain

**Keywords:** fortification, *Lactobacillus pentosus*, olive packing, probiotic vegetables, table olives

## Abstract

Dairy products are currently the main carriers of probiotic microorganisms to the human body. However, the development of new matrices for probiotic delivery is convenient for intolerant to milk (or its derivatives) and those requiring low-cholesterol diet consumers. The present work focused on the fortification of previously fermented green Spanish style olives with the autochthonous putative probiotic bacteria *Lactobacillus pentosus* TOMC-LAB2. The fortification was carried out by inoculating the bacteria into the packing brines using Manzanilla fruits from three different processes: (i) spontaneously fermented (F1), (ii) fermented using *L. pentosus* TOMC-LAB2 as starter (F2), and (iii) spontaneously fermented and then thermally treated (F3). Data showed that all inoculated treatments had higher population levels (5.49, 4.41, and 6.77 log_10_ cfu/cm^2^) than their respective controls (1.66, 4.33, and 0.0 log_10_ cfu/cm^2^, for F1, F2, and F3 treatments, respectively). The presence of *L. pentosus* TOMC-LAB2 on olive surface was confirmed by rep-PCR, with a recovery frequency at the end of the shelf life (200 days) of 52.6, 57.9, and 100.0% for F1, F2, and F3 treatments, respectively. Thus, results obtained in this work show the ability of this microorganism to survive under packing conditions for long period of times as well as to colonize the olive surface which is the food finally ingested by consumers. This opens the possibility for the development of a new and simply probiotic fortified olive product.

## INTRODUCTION

Table olive is one of the most important fermented vegetable of the food industry in the western countries, especially in the Mediterranean basin, with Spain, Turkey, Egypt, Italy, and Greece as the main producers. The International Olive Oil Council (IOC) estimated that the last consolidate balance of table olive production (2011/2012 season) was above 2.4 million tons (International Olive Council [IOC], 2014). Approximately 60% of this amount was processed according to green Spanish-style table olives, which implies a lye treatment of fruits (1.8–2.5% NaOH, w/v), followed by washing and brining (10–11% NaCl, w/v) where fruits undergo a typical lactic acid fermentation (Garrido-Fernández et al., 1997).

Lactic acid bacteria (LAB) exhibit diverse characteristics with potential benefits on human health. Among others, the following favorable effects have been documented: improvement of food digestibility and lactose assimilation, modulation of the immune response, reduction of hypercholesterolemia, prevention of intestinal infections, cancer, food allergies, and constipation (Champagne and Gardner, 2005). To these favorable aspects, we have to add that table olives might also be considered as a functional food itself because of their high content in antioxidant compounds, vitamins, dietary fiber, and anticancer compounds (Garrido Fernández et al., 2001; Reyes-Zurita et al., 2009).

Fermented vegetables are nowadays being considered as a splendid source and vehicle of probiotic microorganisms (Peres et al., 2012). Probiotic vegetables have the potential to attract more consumers who demand functional products, since vegetables provide to those who are intolerant to milk and its derivatives, or require low-cholesterol diets, access to new probiotic formulations (Lavermicocca et al., 2010; Peres et al., 2012). Packed green Spanish-style table olives can be thermally treated for preservation, but they can also be stabilized simply by physicochemical characteristics, being in this case considered as ready-to-eat product directly consumed without any prior cooking. This makes them very convenient carriers of beneficial microorganisms to the human body.

Ranadhera et al. (2010) considered that the type of food carrier plays an essential role in buffering the probiotic during its passage throughout the gastrointestinal tract, regulating their colonization or interacting with other microorganisms to alter functionality. Lavermicocca et al. (2005) used table olives as a vehicle to incorporate exogenous probiotic bacteria species into the human body. Particularly, one strain of *Lactobacillus rhamnosus* was remained invariant until the end of the experiment and showed a good recovery after fermentation. *L. paracasei* IMPC2.1 successfully colonized the olive surface and dominate the natural LAB population until the end of the fermentation (De Bellis et al., 2010), making the product a suitable carrier for its delivery to humans. Recently, Argyri et al. (2013a) and Bautista-Gallego et al. (2013) have isolated diverse LAB strains from table olive processing with promising characteristics for their use as probiotic agent.

Usually, the development of probiotic table olives has been focused on the application of these microorganisms as starter cultures at the onset of fermentation. A recent study showed that the inoculation with multifunctional lactobacilli starters led to a decrease in the *Enterobacteriaceae* populations and to higher LAB and yeast populations as well as to a faster acidification of the brines (Rodríguez-Gómez et al., 2013). Argyri et al. (2014) and Blana et al. (2014) also evaluated with promising results diverse potential probiotic LAB strains, originally isolated from olive fermentation, as starter cultures in table olive fermentation.

On the contrary, the present study is completely novel because is focused on the application of autochthonous putative probiotic microorganisms isolated from olive processing not during fermentation, but at the moment of packing closing with the aim of: (i) the development of a new and easy method for the fortification of table olives with potential probiotic, (ii) studying the survival of bacteria under the packing conditions to determine shelf life of the functional product.

## MATERIALS AND METHODS

### OLIVE PACKING CONDITIONS

The fruits used in the present study were of the Manzanilla variety (*Olea europaea pomiformis*), previously fermented according to the green Spanish-style (Garrido-Fernández et al., 1997). To mimic industrial packing conditions, after 6 months of fermentation (from October 2010 to March 2011), 175 g of olives were introduced into A314 jars (314 mL volume, 75 mm diameter × 103.5 mm high) and covered with 145 mL of new fresh brine. This brine had the adequate concentrations of salt and lactic acid to reach 5.5% NaCl and 0.6% titratable acidity, expressed as lactic acid, in the equilibrium. The final pH was, approximately, 3.8 ± 0.1.

### FORTIFICATION OF OLIVES

Packed olives were fortified with overnight cultures of strain *L. pentosus* TOMC-LAB2 (henceforth LAB2), previously selected because of their potential probiotic characteristics (Bautista-Gallego et al., 2013), ability of adhesion to olive epidermis (Arroyo-López et al., 2012), good performance as starter in previous fermentation trials performed at laboratory scale (Rodríguez-Gómez et al., 2013), and high survival to simulated *in vitro* human digestions (0.74%). Inoculum was grown until early stationary phase and then an aliquot of the suspension was added to the packing brines to reach an initial inoculum level of approximately 6.5 log_10_ cfu mL^-1^. The addition was essayed using three different types of fruits:

(i) F1 treatment, fruits obtained from a spontaneous olive fermentation. It included a control (F1), packaged without LAB addition, and olives fortified with LAB2 strain (F1-F).
(ii) F2 treatment, fruits previously fermented using LAB2 as starter culture. It included a control (F2), packaged without further LAB addition, and olives reinforced with the same strain (F2-F) at packing closing.
(iii) F3 treatment, fruits spontaneously fermented and then subjected to a thermal treatment by immersion in a water bath at 85°C for 5 min. It included a control (F3), packaged without further addition of LAB, and fruits fortified with LAB2 strain after cooling of brines (F3-F).

Each treatment was carried out in duplicate, making a total of 12 packing containers which were kept during the entire process at the Instituto de la Grasa pilot plant (CSIC, Seville, Spain) at room temperature (variable from 18°C in March/2011 to 31°C September/2011). The experiment was monitored for 200 days.

### MICROBIOLOGICAL ANALYSES OF FRUITS

Olive samples, or their decimal dilutions, were plated using a Spiral Plating System model dwScientific (Don Whitley Scientific Ltd., Shipley, UK) on the media described below at 0, 38, 80, and 200 days of packing. Plates were counted using a CounterMat v.3.10 (IUL, Barcelona, Spain) image analysis system. *Enterobacteriaceae* were counted on VRBD (Crystal-violet Neutral-Red bile glucose)-agar (Merck, Darmstadt, Germany), LAB on MRS (de Man, Rogosa and Sharpe)-agar (Oxoid) supplemented with 0.02% (w/v) sodium azide (Sigma, St. Louis, MO, USA), and yeasts on YM (yeast-malt-peptone-glucose) agar (Difco^TM^, Becton and Dickinson Company, Sparks, MD, USA) supplemented with oxytetracycline and gentamicin sulfate as selective agents for yeasts. Plates were incubated at 37°C for 24 h (*Enterobacteriaceae*) or 30°C for 48 h (yeasts and LAB).

To determine the number of microorganisms adhered to the olive epidermis, the protocol developed by Böckelmann et al. (2003) was slightly adapted to the specific characteristics of table olives. Briefly, two fruits from each packing container were randomly taken at different sampling times and washed for 1 h with 250 mL of a sterile PBS buffer solution (8.0 g L^-1^ NaCl, 0.2 g L^-1^ KCl, 1.44 g L^-1^ Na_2_HPO_4_, 0.24 g L^-1^ KH_2_PO_4_, pH finally adjusted to 4.7 with HCl 1 M) to remove non-adhering cells. Then, olives were transferred to 50 mL of a PBS solution added of the following enzymes: 14.8 mg L^-1^ lipase (L3126), 12.8 mg L^-1^ β-galactosidase (G-5160) and 21 μL L^-1^ α-glucosidase (G-0660) (Sigma–Aldrich, St. Louis, MO, USA). To achieve biofilm disintegration and removal of the adhered cells, the fruits were incubated at 30°C in this enzyme cocktail with slight shaking (150 rpm). After 12 h, the olives were removed and the resulting suspension was centrifuged at 9,000 × *g* for 10 min at 4°C. Finally, the pellet was re-suspended in 2 mL of PBS and spread onto the different culture media described above. Olive microbial counts were expressed as log_10_ cfu/cm^2^, using the formula of a prolate spheroid for the approximate calculus of olive surface from the longitudinal and transverse axes of fruits (Weisstein, 2013). For the Manzanilla fruits used in the present study, the average area was 10.99 ± 1.01 cm^2^ and the weight 4.08 ± 0.46 g.

### CHARACTERIZATION OF THE LACTIC ACID BACTERIA POPULATION

**FIGURE 1:**
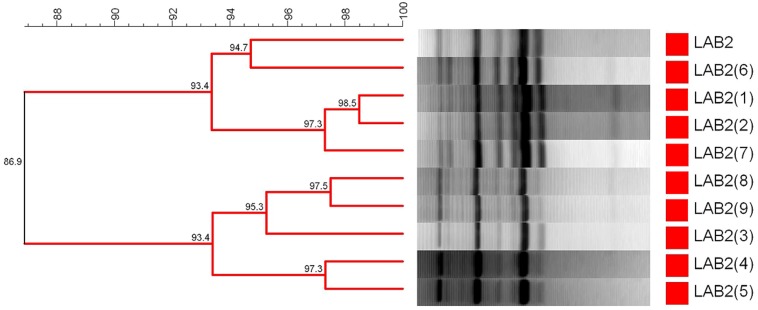
**Dendrogram generated after cluster analysis of the digitalized fingerprints of *L. pentosus* TOMC-LAB2 obtained from 10 different running to determine the repeatability of the rep-PCR technique (86.9%) with primer GTG_**5**_.** Clustering parameters: 0.5% optimization and 0.0 % curve smoothing.

For characterization of the lactobacilli population, repetitive bacterial DNA element fingerprinting analysis (rep-PCR) with primer GTG_5_ was followed using the protocol described in Gevers et al. (2001). The PCR reaction in a final volume of 25 μL contained: 5 μL of 5x MyTaq reaction buffer (5 mM dNTPs and 15 mM MgCl_2_), 0.1 μL of My Taq DNA polymerase (Bioline reactives, UK), 1 μL GTG_5_ primer (25 μm), 13.9 μL desionized H_2_0, and 5 μL DNA (∼20 ng/μL). PCR amplification was carried out in a thermal cycler (Master Cycler Pro, Eppendorf) with the following program: 95°C for 5 min as initial step, plus 30 cycles at: (1) 95°C for 30 s, (2) annealing at 40°C for 30 s, and (3) 65°C for 8 min, with a final step of 65°C for 16 min to conclude the amplification. This methodology was used to determine the recovery frequency of the inoculated strain at the end of packing. Firstly, the repeatability of the technique was determined using the LAB2 strain as internal control, obtaining a 86.9% ± 3.4% of similarity for this bacteria in ten different gels (**Figure 1**). Then, diverse isolates were randomly picked from each treatment at the end of experiment (200 days), making a total of 88 lactobacilli to analyze. They were named with the name of treatment (F1, F2, or F3) and with a “F” in the case of fortified/reinforced olives. Their pattern profiles of bands (from 100 up to 3,000 bp) were compared with the strain used to fortify the treatments (LAB2). For this purpose, PCR products were electrophoresed in a 2% agarose gel and visualized under ultraviolet light by staining with ethidium bromide. The resulting fingerprints were digitally captured and analyzed with the BioNumerics 6.6 software package (Applied Maths, Kortrijk, Belgium). The similarity among digitalized profiles was calculated using the Pearson product-moment correlation coefficient. Dendrograms were obtained by means of the Unweighted Pair Group Method using Arithmetic Average (UPGMA) clustering algorithm and the automatic calibration tool for the determination of the optimization and curve smoothing parameters.

### STATISTICAL ANALYSIS

Analysis of variance was performed by means of the one-way ANOVA module of Statistica 7.1 software to check for significant differences among treatments. For this purpose, a *post hoc* comparison was applied by means of the Scheffé test.

## RESULTS AND DISCUSSION

After fermentation (6 months), three different types of fruits where placed in new fresh brine and inoculated with a putative probiotic bacteria (LAB2) just before closing the containers. Initial physicochemical conditions (pH, salt, free, and combined acidity) were stable during the whole period of packing (data not shown). Below, the main results obtained for the changes in the microbial populations and the presence of the inoculated strain after 200 days of shelf life are shown.

### EVOLUTION OF MICROBIAL POPULATIONS THROUGH PACKING

Diverse works have reported that the survival of microorganisms through table olive packing is considerable. Argyri et al. (2013b) showed that microbial population after 40 days of Halkidiki green table olive packing was above 7 and 5 log_10_ cfu g^-1^ for LAB and yeasts, respectively. Bautista-Gallego et al. (2011) reported that after 60 days of Aloreña directly brined packing, the counts for LAB and yeasts were above 5 and 6 log_10_ cfu mL^-1^, respectively. For this reason, it can be expected that probiotic bacteria could also survive for long period of time under packing conditions, and thereby, be used for fortification purposes.

**FIGURE 2:**
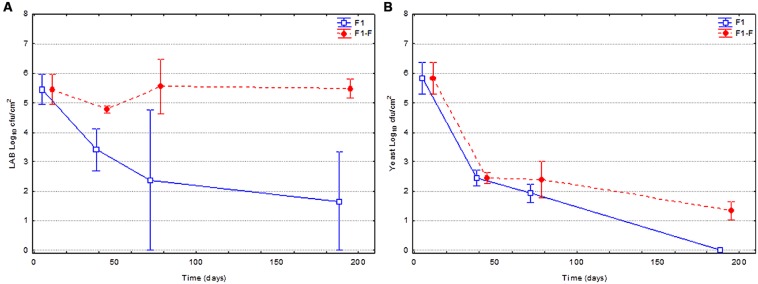
**Changes in the LAB **(A)** and yeast **(B)** populations on olive surface during shelf life.** Control (F1) corresponded to just packaged spontaneously fermented fruits, while fortified (F1-F) were those inoculated with *L. pentosus* TOMC-LAB2 strain.

**Figure 2** shows the evolution of the LAB and yeasts population on olive surface for fruits spontaneously fermented (F1) and fortified (F1-F) with LAB2 strain at the moment of packing. A clear and statistical significant difference between the changes in both LAB populations in the two types of treatments was noticed (**Figure 2A**). In the case of F1 fruits, a marked decline of population was observed through packing, while in fortified fruits (F1-F), LAB counts were always higher and practically constant during the shelf life. Thereby, at the end of the studied period (200 days), the population for fortified fruits was 5.5 against 1.7 log_10_ cfu/cm^2^ for non-fortified fruits. In the case of yeast population (**Figure 2B**), the evolution between both treatments was similar but with slight differences. A marked decline of yeast population was observed through packing in both cases, from the initial 5.8 to 1.3 (F1-F) or 0.0 (F1) log_10_ cfu/cm^2^. Then, yeast survival was also slightly (but statistical significant) favored by the addition of the bacteria.

**FIGURE 3:**
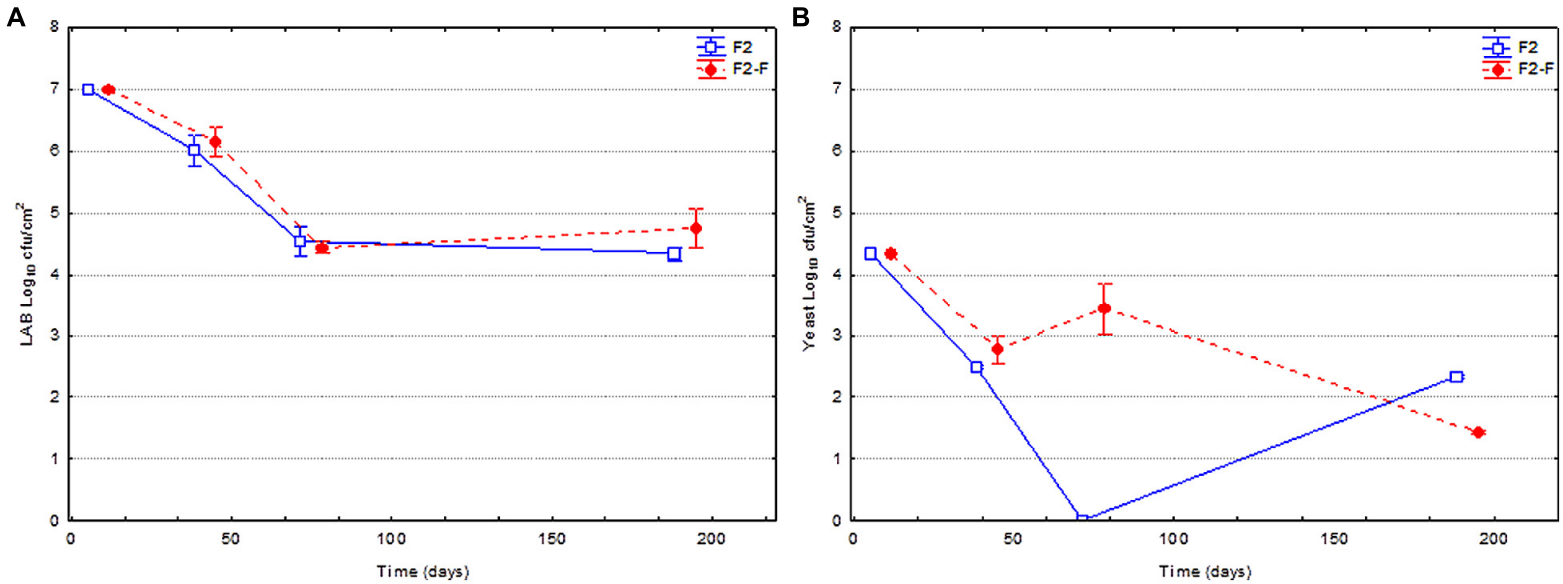
**Changes in the LAB **(A)** and yeast **(B)** populations on olive surface through shelf life.** Fruits used in this experiment had been previously fermented using *L. pentosus* TOMC-LAB2 as starter culture, and just packaged (F2) or reinforced (F2-F) with the same strain at the moment of closing containers.

**Figure 3** shows the evolution of the LAB and yeast populations on olive surface for fruits previously fermented using LAB2 as starter culture, packaged just as they were processed (F2) or reinforced with the same strain just before closing of containers (F2-F). The changes in the LAB population was very similar (no statistical differences) between reinforced and non-reinforced fruits, with a progressive reduction from the initial 7.0 to around 4.4 log_10_ cfu/cm^2^ at 80 days. Later on, the population remained, in practice, constant until the end of experiment. The evolution of the yeast population was very similar as in the previous case, with a clear decline through packing period (from the initial 4.3 to around 2.0 log_10_ cfu/cm^2^ at the end of study), although a higher population was observed during most of the shelf life.

**FIGURE 4:**
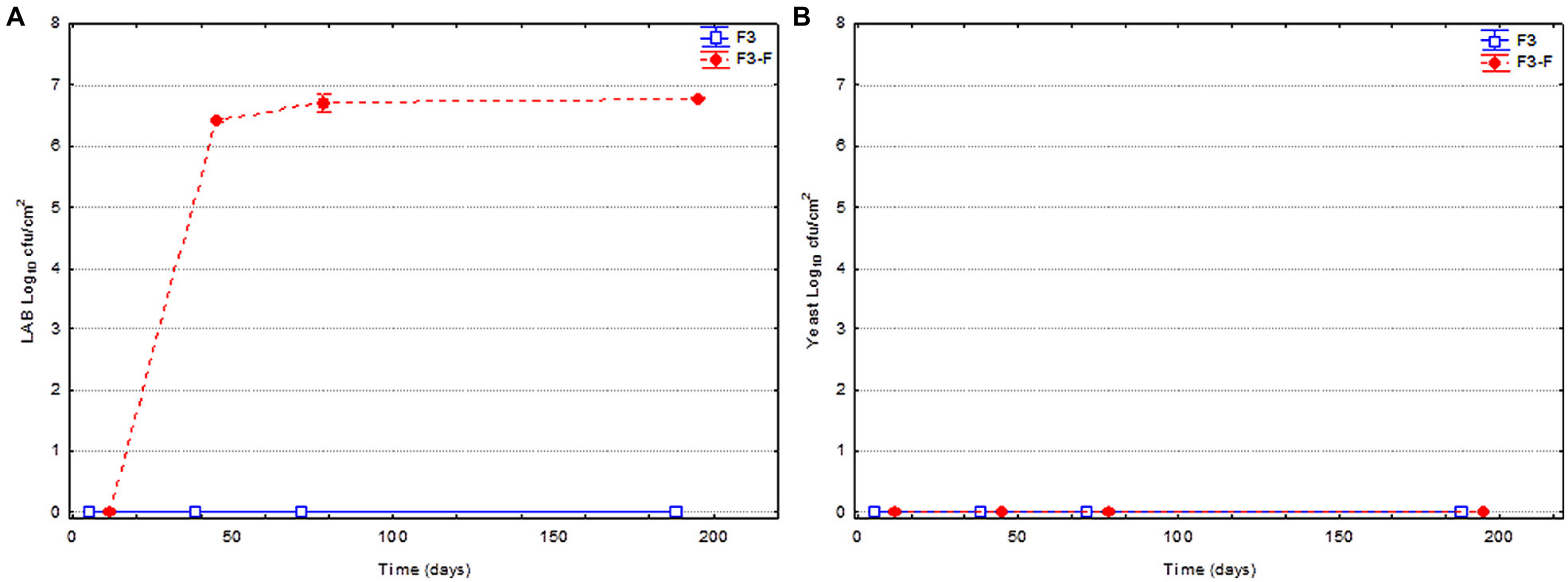
**Changes in the LAB **(A)** and yeast **(B)** populations on olive surface through shelf life.** Olives were spontaneously fermented and treated with heat (85°C during 5 min). Part of fruits were just packed after thermal treatment (F3), while others were fortified (F3-F) with *L. pentosus* TOMC-LAB2 strain at the moment of closing containers.

**Figure 4** shows the evolution of the LAB and yeast populations on olive surface for fruits spontaneously fermented and thermally treated before packing. As commented, some fruits were packed without LAB addition (F3), while others were fortified with LAB2 strain (F3-F). Apparently, the thermal treatment (85°C for 5 min) was very efficient for killing the vegetative cells of both LAB and yeasts adhered to the olive surface, and no microbial cells were detected, except in the treatments inoculated with LAB2 strain (**Figure 4A**). In this case, the LAB added to the brine was able to colonize the olive surface in less than 38 days. In fact, the population increase from 0.0 to approximately 6.8 log_10_ cfu/cm^2^ was very fast and persisted up to the end of the shelf life.

Finally, *Enterobacteriaceae* were not detected in any of the packing treatments assayed through shelf life. This is in agreement with the low pH obtained in the packing conditions (pH < 4.0), which is a very inhibitory factor for the survival of this group of microorganism (Garrido-Fernández et al., 1997), but also due to the lack of appropriate nutrients because the sugars were previously exhausted during fermentation process (data not shown).

Therefore, data obtained in this work show the ability of the LAB2 strain to move from packing brine onto the fruits and to colonize the olive surface, which was especially evident in the case of F3-F treatment. This is in agreement with results obtained recently by Arroyo-López et al. (2012) and Domínguez-Manzano et al. (2012) who demonstrated that diverse *L. plantarum* and *L. pentosus* strains were able to establish polymicrobial communities with yeasts on the surface of olives processed according to green Spanish-style. Peres et al. (2012) mention diverse types of technological processes (microencapsulation, vacuum, and immersion impregnation, cell immobilization, etc.) to fortify plant matrices with probiotic microorganism. In this work, the simply preparation of a packing brine with a concentration of ∼ 6.5 log_10_ cfu mL^-1^ of LAB2 strain, resulted in a single and easy method for the fortification of olives with the desire bacteria by means of a surface adhesion mechanism.

### PRESENCE OF THE INOCULATED STRAIN AT THE END OF SHELF LIFE

The presence of the inoculated LAB2 strain at the end of packing was corroborated by molecular methods (rep-PCR) using a total of 88 lactobacilli isolates obtained from F1, F2, F3, and their respective fortified/reinforced treatments.

**FIGURE 5:**
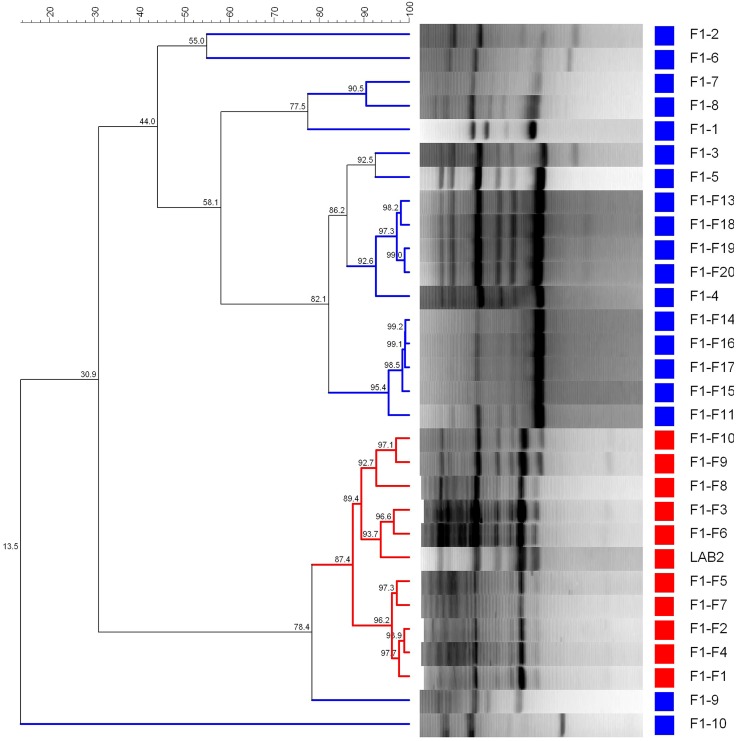
**Dendrogram generated after cluster analysis of the digitalized rep-PCR fingerprints with primer GTG_**5**_ of 29 lactobacilli isolates obtained from fruits spontaneously fermented.** Part of fruits were just packaged (F1, control) or fortified with *L. pentosus* TOMC-LAB2 strain (F1-F), while 1–20 is the number for the isolates obtained within each treatment. Clustering parameters: 0.5% optimization and 0.0% curve smoothing.

**Figure 5** shows the dendrogram generated using the patterns profile of 29 LAB isolates randomly obtained from epidermis of F1 (10) and F1-F (19) fruits plus the inoculated LAB2 strain. The cluster analysis showed that 10/19 isolates obtained from the fortified F1 treatment shared a 87.4% similarity with the inoculated LAB2 strain, which was above repeatability of the technique (86.8%). Thereby, LAB2 strain showed in F1-F treatment a recovery frequency of 52.6%, while 9/19 isolates (47.4%) where grouped in different clusters together with many of the isolates obtained from the spontaneous and non-fortified F1 treatment.

**FIGURE 6:**
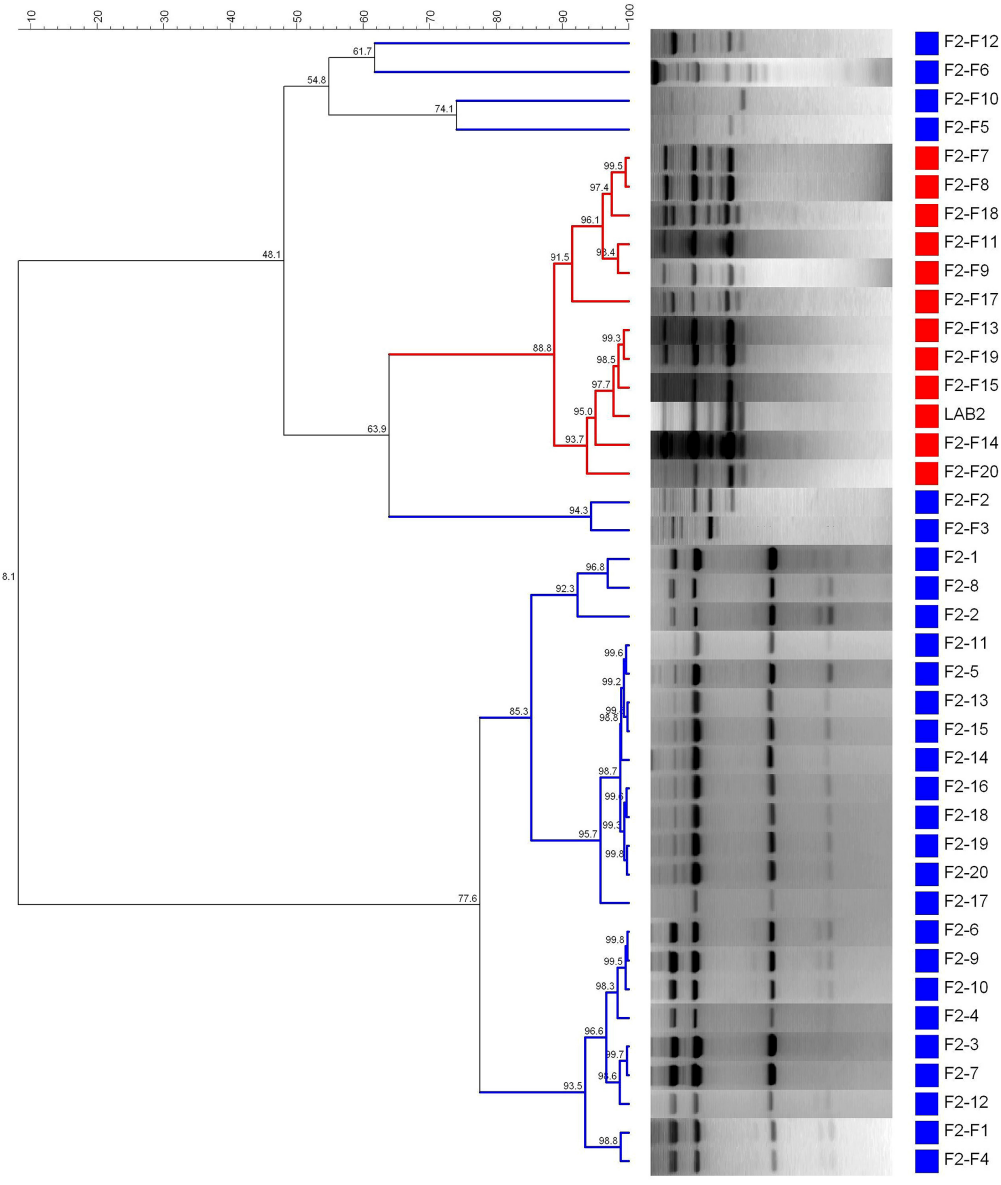
**Dendrogram generated after cluster analysis of the digitalized rep-PCR fingerprints with primer GTG_**5**_ of 39 lactobacilli isolates obtained from fruits previously fermented using *L. pentosus* TOMC-LAB2 as starter culture**. Part of fruits were just packaged (F2, control) or reinforced with the same strain at packing closing (F2-F), while 1–20 is the number for the isolates obtained within each treatment. Clustering parameters: 0.5% optimization and 5.0% curve smoothing.

**Figure 6** shows the dendrogram generated using the patterns profile of 39 lactobacilli isolates randomly obtained from epidermis of F2 (20) and F2-F (19) fruits plus the inoculated LAB2 strain. The cluster analysis showed that 11/19 isolates obtained from the reinforced F2-F treatment shared a 88.8% similarity with the inoculated LAB2 strain, which again was above repeatability of the technique. Thus, LAB2 strain showed in the reinforced treatment a recovery frequency of 57.9% at 200 days of packing, while 8/19 isolates (42.1%) where grouped in different clusters with many of the isolates obtained from the non-reinforced F2 treatment. It is remarkable to verify that, in the case of no inoculation, it was not possible to recover the profile of the LAB2 strain from the F2 treatment even when this microorganism was used as starter culture at the onset of fermentation. Thus, a reinforcement step with this strain at the beginning of packing to assure its presence during shelf life it is always advisable.

**FIGURE 7:**
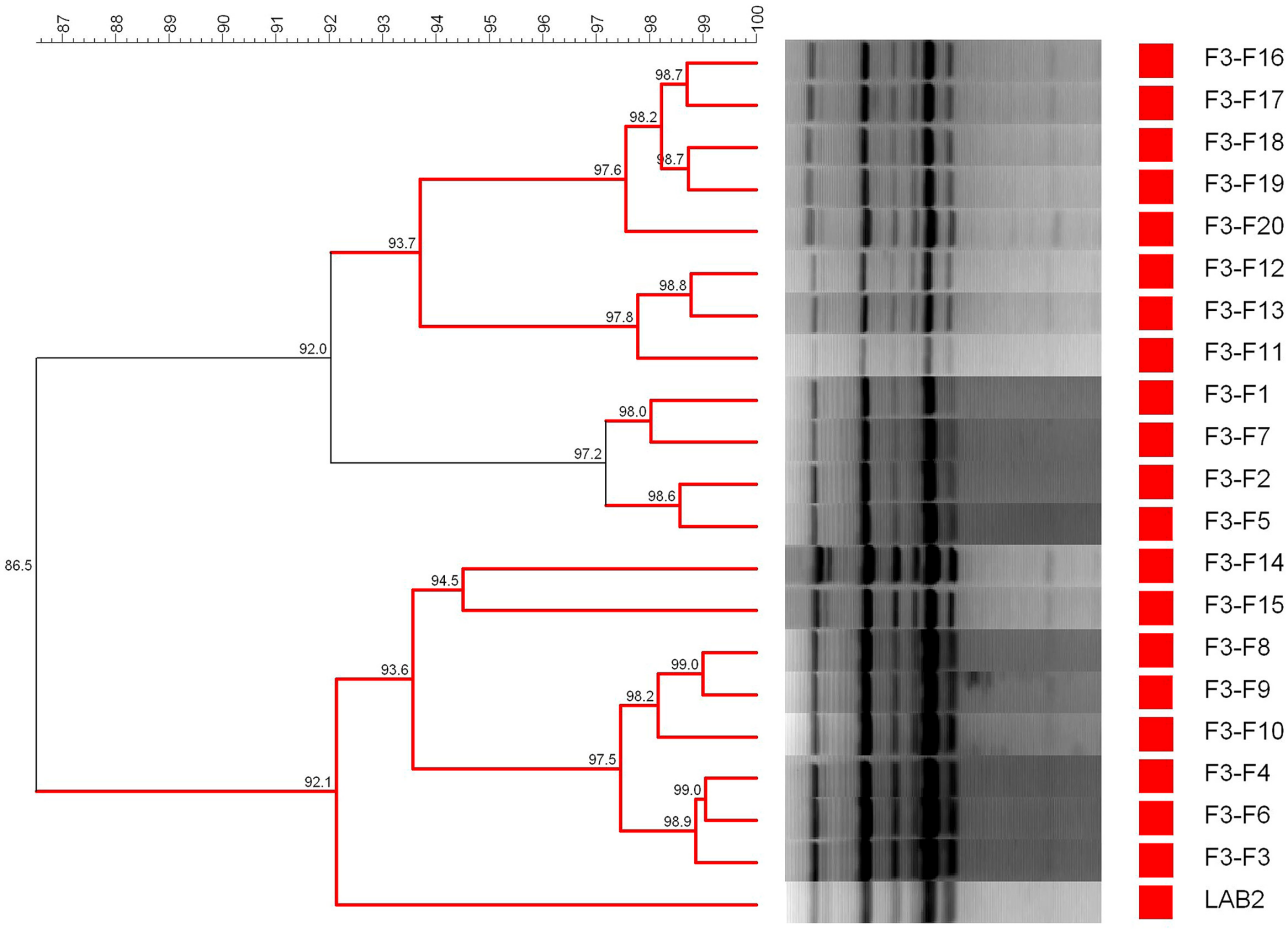
**Dendrogram generated after cluster analysis of the digitalized rep-PCR fingerprints with primer GTG_**5**_ of 20 lactobacilli isolates obtained from fruits spontaneously fermented and then thermally treated (85°C for 5 min).** Information corresponds to only F3-F treatment (fruits fortified with *L. pentosus* TOMC-LAB2 strain) since no growth was detected in F3, while 1–20 is the number for the isolates obtained in the treatment. Clustering parameters: 0.5% optimization and 0.0% curve smoothing.

Finally, **Figure 7** shows the dendrogram generated using the patterns profile of 20 lactobacilli isolates randomly obtained from olive epidermis of F3-F treatments plus the inoculated LAB2 strain. In this case, because of the thermal treatment, no LAB isolates were obtained from F3 treatment. The cluster analysis showed that 20/20 isolates obtained from the F3-F treatment shared a 86.5% similarity with the inoculated LAB2 strain, which is in the limit of repeatability of the technique. Thereby, LAB2 strain showed in the fortified treatment a recovery frequency of 100.0% at 200 days, which is logical because, in this case, the survival of other microorganism was prevented by the thermal treatment.

Taking into account the average surface values obtained for the Manzanilla fruits used in the present study (11 cm^2^), a total of ∼ 10^8^ LAB cells could be ingested by consumers who eat only one fortified olive with LAB2 strain after thermal treatment. Data could also be converted to cfu g^-1^ taking into account the average weight of fruits (4.08 g). Probiotic microorganisms have to be taken regularly and to sufficiently high levels (at least 10^6^ cfu mL^-1^ per daily dose) to avoid washout, and to assure that their benefits will be accrued in a sustained manner (Peres et al., 2012). The Spanish Association of Processors and Exporters (ASEMESA, 2014) considers 7 olives (about 25 g of olive flesh) as a satisfactory daily intake. Olives thermally treated and fortified with LAB2 strain clearly exceed the number of recommended probiotic microorganism. Pasteurization of Spanish green table olives using glass and tin as packing material is usually carried out by industry to enlarge the shelf life of the product using a minimum amount of preservatives (Garrido-Fernández et al., 1997). Thus, this type of product could be a convenient carrier of probiotic to the human body because of the high number of microorganisms that potentially could be ingested by consumers with a larger shelf life compared to dairy product.

## CONCLUSION

Data obtained in this work show that fortification of table olives with *L. pentosus* TOMC-LAB2 is possible because of the considerable survival of this microorganism during shelf life at room temperature without chill chain. Among the different types of fortification assayed, a thermal treatment after fermentation to eliminate microorganism and later inoculation with the bacteria is desired because of the high counts and recovery frequency of the added LAB bacteria obtained at the end of the packing period. However, sensorial studies should be also carried out to determine the influence of the thermal treatment or bacteria growth on the sensory profile of packaged olives.

## Conflict of Interest Statement

The authors declare that the research was conducted in the absence of any commercial or financial relationships that could be construed as a potential conflict of interest.
